# Divide and Conquer: Strategies in Singapore to Manage a Nuclear Medicine Department During COVID-19

**DOI:** 10.2967/jnmt.120.244855

**Published:** 2020-06

**Authors:** Wei Y. Tham, Aaron K.T. Tong, Kelvin S.H. Loke, Liyi Chio, Gabriel K.Y. Lim, Xin Y. Seah, David C.E. Ng, Sean X.X. Yan, Winnie W.C. Lam

**Affiliations:** Department of Nuclear Medicine and Molecular Imaging, Singapore General Hospital, Singapore

**Keywords:** epidemic, COVID-19 infection, infection control

## Abstract

The COVID-19 outbreak was declared a public health emergency of international concern by the World Health Organization on January 30, 2020. Since then, the virus has spread to affect more countries worldwide. During this period, our nuclear medicine department at Singapore General Hospital segregated our staff and patients by time, by space, or both, to minimize contact and prevent spread of the virus. Necessary changes to our clinical practices and stricter infection control measures were also enforced. We share our personal experience in managing a nuclear medicine department during this epidemic.

In December 2019, China reported several cases of pneumonia of uncertain etiology linked to the Hunan seafood market in Wuhan. These cases were was later found to be due to a novel coronavirus (1). Bats appear to be the reservoir of the virus; however, the intermediary host is not yet known (2). Symptoms include fever, cough, and breathlessness, although some patients who tested positive via reverse-transcription polymerase chain reaction were asymptomatic. Transmission between humans has been confirmed, although the transmission routes are still unclear. The outbreak was declared a public health emergency of international concern by the World Health Organization on January 30, 2020. On February 28, the World Health Organization raised the highest alert for risk of spread of the disease and risk of impact from it (3).

The first patient detected with coronavirus disease 2019 (COVID-19) in Singapore was admitted to the Singapore General Hospital on January 23, 2020 (4). Initially, all cases were imported. However, in early February, community cases (positive patients who did not have any recent travel to mainland China or links to the previous cases) were detected. The Ministry of Health implemented additional precautionary measures, such as regular temperature and symptom screening at workplaces, as well as cancellation or deferment of large-scale events, to minimize the risk of further transmission of the virus in the community (4). By March 9, 2020, there were 160 confirmed cases in the country, with 10 critically ill.

Singapore General Hospital is the largest tertiary hospital in Singapore. It also houses the largest nuclear medicine department in the country. Our department currently has 1 PET/CT scanner, 3 γ-cameras, 1 SPECT/CT scanner, 1 ultrasound machine, 1 treadmill for cardiac stress testing, and 1 bone densitometer. We provide inpatient and outpatient services and both diagnostic and therapeutic nuclear medicine services. Our department also provides satellite nuclear medicine services for some of the other hospitals in our health-group cluster. The challenges arise from the fact that nuclear medicine is a diverse discipline involving many types of staff, including laboratory staff, nuclear medicine technologists, administrative staff, nurses, and doctors.

To deal with this national emergency, we put measures in place that minimize the risk of disease transmission. Work procedures in our institution were reviewed. We discussed and then adopted various strategies to modify the usual practices in the department yet maintain normal functioning as much as possible.

## SEGREGATION AMONG PATIENTS AND STAFF

Previous experience with the severe acute respiratory syndrome outbreak in Singapore in 2003 taught us that disease transmission takes place within the hospital. Radiology-related infections were seen in staff members and in visitors or outpatients to the radiology department (5). Therefore, segregation is needed to differentiate people of different infection risks.

**FIGURE 1.**
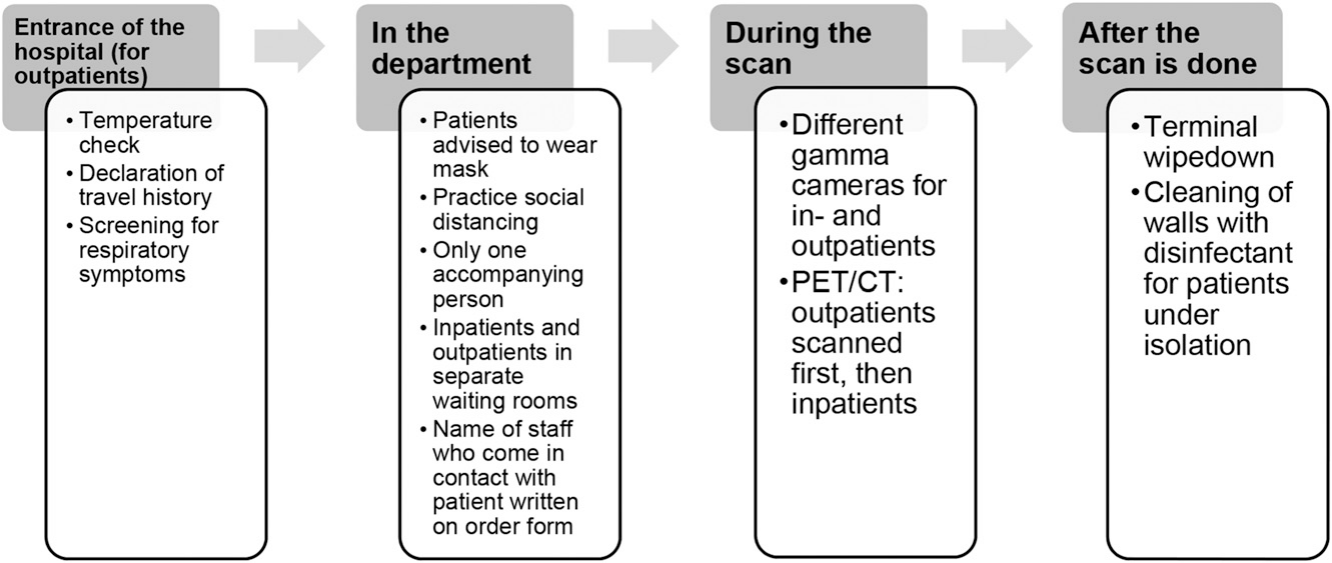
Procedural flowchart.

Patients were segregated by time, by space, or both according to the likelihood of transmission. At the entrance to the hospital, outpatients were screened for recent travel and symptoms before being allowed to enter the department. Temperature checks were done. Only 1 accompanying person was allowed for each patient. Two of the γ-cameras were dedicated to inpatients, and the others were used for outpatients. As we have only 1 PET/CT scanner, outpatient scans were done before inpatient scans to minimize contact between the different groups of patients. Patients under isolation were scanned as the last cases of the day, when there would be fewer patients in the department. There were also designated waiting rooms for inpatients so they would not be in physical contact with outpatients. A procedural flowchart is presented in Figure 1.

**FIGURE 2.**
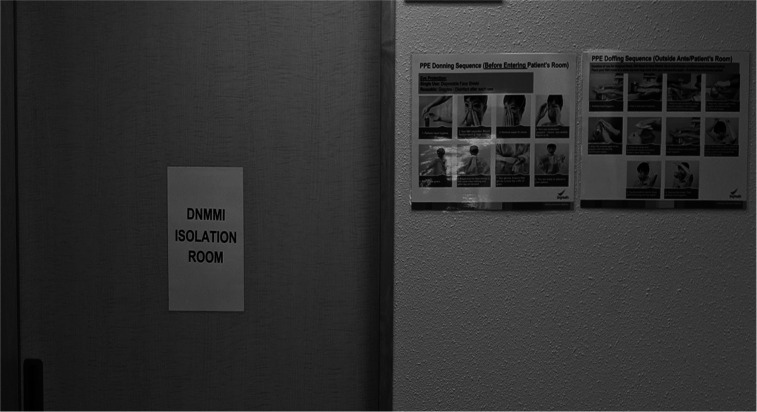
Photograph of isolation room in our nuclear medicine department. DNMMI = department of nuclear medicine and molecular imaging.

One of the rooms was set aside as a designated isolation room (Fig. 2). Patients suspected to have COVID-19 were escorted to this room until further assessment could be done (patients would wear a face mask while those escorting the patients would don personal protective equipment [PPE]). Management decisions, such as whether admission was required, were made with the advice of an infectious disease specialist. Time is also of importance. Patients were managed promptly, and as safely as possible, so that they spent less time near other patients.

Staff were divided into teams and encouraged to minimize interaction among one another to prevent contamination. The nuclear medicine technologists were assigned fixed rooms in which to work. Doctors were also segregated. Cross-institutional work was stopped or reduced to a workable minimum, and one physician was assigned to one of the other hospitals for the time being. Fellow colleagues from other hospitals helped with some of the radionuclide injections, such as sentinel node injections, and the scans were reported remotely. Nuclear medicine physicians within Singapore General Hospital were split into 2 teams. One had patient contact, and the other reported scans remotely from another office building. Duties were exchanged every 2 wk to prevent physician burnout.

New applications for overseas travel to high-risk countries were discouraged, and some previously booked travel plans were cancelled in a display of solidarity and in case more staff were needed. Relevant information-technology platforms, including the use of secured social media such as Workplace by Facebook and Tiger Connect, were adopted to facilitate communication among staff (6).

Appointments for patients from high-risk countries such as China were postponed when possible. Otherwise, these patients were expected to fulfil a specified home quarantine of 14 d if they were symptomatic and came from affected countries. The same expectation applied to staff who returned from high-risk areas.

During this period, all student internships and clinical postings were suspended to avoid unnecessary exposure. Doctors under training were not allowed to move to other hospitals for other rotations. Multidisciplinary tumor boards and discussions would usually physically bring together specialists from various disciplines to discuss cases and were generally discouraged; many moved to online discussions via video conferencing.

## CHANGES TO CLINICAL PRACTICE

Some modifications were done for scans. For example, aerosolization was stopped for the ventilation part of the ventilation–perfusion lung scans, and we made do with perfusion-only scans when necessary. Myocardial perfusion imaging was done mainly with pharmacologic stress when possible. A 1-d rest–stress protocol was preferred over our usual 1- or 2-d stress–rest protocol, as the former would minimize patient transfers and duration of exposure.

It has been suggested that COVID-19 pneumonia may show abnormalities on a chest CT scan during the subclinical phase of the disease. Special attention was paid to early review of the lungs on PET/CT or SPECT/CT images to identify any patients with possible ground-glass changes—a finding that may indicate early infection (7,8). Patients who had lung findings possibly secondary to COVID-19 infection were quickly isolated and admitted for further investigations after consultation with the primary physician and infectious disease expert.

## CONTACT TRACING

Contact tracing is an important part of trying to curb spread of the virus (9). In estimating the probability of detection in other countries, Niehus et al. (10) published a paper estimating the probability of detecting an imported case in other countries using Singapore as the standard, as they considered the detection rate in Singapore to reflect the highest surveillance capacity among all locations affected by the virus. We had to play our part in efforts to trace contacts. In our department, extra measures were put in place to facilitate contact tracing, with special care taken to note which personnel attended to each patient.

## INFECTION CONTROL

**FIGURE 3.**
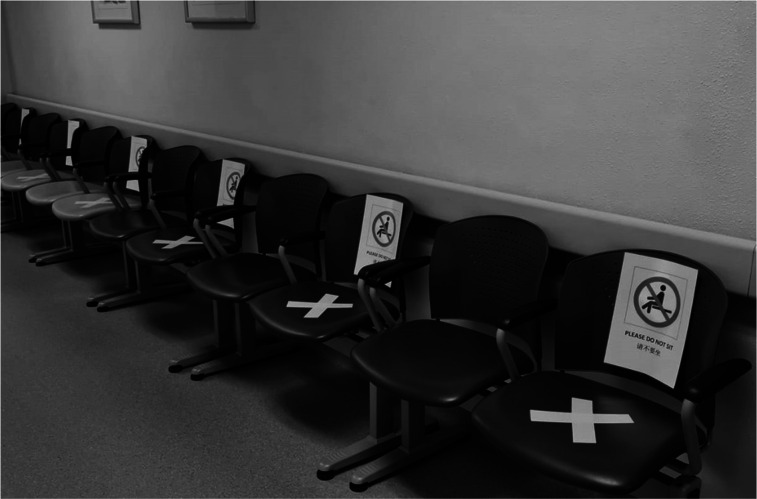
Photograph of waiting area encouraging social distancing in our nuclear medicine department.

It is important to protect our frontline staff who continue to have patient contact. More stringent infection control measures were taken, and refresher courses for infection control were conducted. Staff in the clinical areas or at the counters were asked to wear surgical face masks at all times. Hand hygiene techniques were emphasized. To minimize the human traffic in the department, patients who had nonurgent follow-up clinic appointments were contacted, and appointments were postponed if the patients were deemed medically stable. Arrangements were made for prescriptions or medications to be delivered to their address to ensure they had a sufficient supply until the next appointment. Staff temperature was recorded twice a day. Staff with a temperature of 37.5°C or more, as well as those who were unwell and had respiratory symptoms, had to seek medical attention in the staff clinic or at the emergency department. They were granted medical leave till they were fit to return to work. Depending on their risk of exposure, they were possibly tested for COVID-19. Each staff member was also fitted with an N95 mask or a powered air-purifying respirator should the need to use it arise, such as when coming into contact with patients from isolation wards or during aerosol-generating procedures. Social distancing, or limiting the social contact and increasing the physical distance between people, was deemed an important policy to reduce the risk of transmission of the virus (Fig. 3).

Senior management and designated infection control officers within each department embarked on a daily walkabout exercise to audit all the different clinical areas in the division. These walkabouts were done by teams of 2 or 3 personnel with at least 1 external auditor, all of whom practiced safe social distancing from one another. Observations were made and photos taken, with reports generated daily to quickly correct any infection control lapses or any safety or workflow issues.

Fomites have been widely implicated as a cause of transmission (11). The imaging table was covered with bedsheets, which were changed after every patient to prevent direct contact between the scanner and the patient. During positioning, the linen and blankets were also placed between the safety straps and the patient. Patients were imaged with their mask on to prevent droplets from contaminating the equipment. The surroundings were cleaned as frequently as possible to provide a safe environment for the staff and patients. The walls of the imaging rooms were disinfected after imaging of patients from isolation wards. Terminal cleaning, which was already being done after every patient, was further emphasized during this period.

## STAFF MORALE

The psychologic well-being of the people in the department must be looked after during this stressful period. The hospital sent frequent updates about the situation and the condition of the patients to keep everyone abreast of the latest information. Words of encouragement from the general public and the hospital administration were shared to acknowledge the hard work and efforts of staff. Helplines providing psychologic support were also made available.

We recognize that it is our responsibility to create a safe environment for our staff and patients. Creating such an environment requires that all involved engage in a cooperative team effort against spread of the virus.

## CONCLUSION

We have described our personal experience in managing a nuclear medicine department during the COVID-19 epidemic. The take-home message is to screen patients at the entrance, segregate patients with different infection-risk profiles, divide staff into smaller groups, emphasize hand hygiene and infection control, practice social distancing, modify scans to minimize risk to staff, and have protocols in place to manage patients with possible infection. As of today, we have not detected any transmission of the virus among our patients or staff—a testament to the effectiveness of the measures taken. Such operational measures are important to help control transmission of the virus while minimally disrupting the necessary functioning of an established nuclear medicine department.

## DISCLOSURE

No potential conflict of interest relevant to this article was reported.
